# Association between serum uric acid level and metabolic syndrome components

**DOI:** 10.1186/s40200-015-0200-z

**Published:** 2015-09-14

**Authors:** Sara Nejatinamini, Asal Ataie-Jafari, Mostafa Qorbani, Shideh Nikoohemat, Roya Kelishadi, Hamid Asayesh, Saeed Hosseini

**Affiliations:** Non-Communicable Disease Research Center, Endocrinology and Metabolism Population Sciences Institute, Tehran University of Medical Sciences, Tehran, Iran; Department of Community Medicine, School of Medicine, Alborz University of Medical Sciences, Baghestan Boulevard, Karaj, Iran; Department of Cardiology, Mazandaran University of Medical Sciences, Sari, Iran; Department of Pediatrics, Child Growth and Development Research Center, Research Institute for Primordial Prevention of Non-communicable Disease, Isfahan University of Medical Sciences, Isfahan, Iran; Department of Medical Emergency, Qom University of Medical Sciences, Qom, Iran; Endocrinology and Metabolism Research Center (EMRC) Institute, Dr Shariati Hospital, Tehran University of Medical Sciences, North Kargar St, Tehran, Iran

**Keywords:** Uric acid, Metabolic syndrome, Insulin resistance, Body composition

## Abstract

**Background:**

Serum uric acid levels is reported to be associated with a variety of cardiometabolic risk factors; however, its direct association with metabolic syndrome (MetS) remains controversial. Thus, we examined the association of serum uric acid concentrations with the MetS components.

**Methods:**

MetS was defined according to the National Cholesterol Education Program (NCEP) criteria. This case–control study comprised 101 non-smoking individuals (41 in the MetS group and 60 in the non-MetS group). Blood pressure, fasting plasma glucose, insulin, HOMA-IR, lipid profiles, uric acid, and anthropometric measures were determined, and body composition was assessed by using bioelectrical impedance analysis (BIA).

**Results:**

After adjustment for confounding factors, serum uric acid was significantly higher in MetS group than non-MetS group (5.70 ± 1.62 vs 4.97 ± 1.30 mg/dL, respectively, *P* = 0.001). After controlling for age, sex and body mass index in partial correlation analysis, uric acid was positively correlated with triglycerides, and negatively with HDL-C. In multiple logistic regression analysis, every 1 mg/dl elevation in the serum uric acid level increased the risk of MetS approximately by 2-folds (OR: 2.11, 95 % CI: 1.30-3.41).

**Conclusion:**

This study showed that those individuals with MetS have higher uric acid levels; the association of uric acid and MetS components supports that it might be an additional components of MetS.

## Introduction

Metabolic syndrome (MetS) is defined by a cluster of risk factors, including obesity, dyslipidemia, hypertension and insulin resistance [1]. When occurring together, they increase the risk of developing cardiovascular disease (CVD) and diabetes [2]. Previous studies have shown that the defined MetS risk factors cannot explain all CVD events observed in these subjects. Therefore, several other risk factors such as inflammatory markers, microalbuminuria, hyperuricemia and disorders of coagulation have been debated to be included in the MetS definition [3–6]. The prevalence of MetS is increasing worldwide including Asian countries [7], and a high prevalence of MetS (30.1 %) has been reported in Iran [8].

Serum uric acid is a final enzymatic product of purine metabolism in humans, and it is suggested that hyperuricemia is associated with MetS, and they may have common pathophysiology [9]. In addition to MetS, elevated concentrations of uric acid are associated with a variety of cardiovascular conditions [10]. However, the association of uric acid and MetS remains controversial and limited experience exists on this relationship. Given the high prevalence of MetS in Iranian population, in the present study we evaluated the association of serum uric acid levels and MetS components in the present study.

## Methods

This case–control study was conducted from February to July 2011 on 105 individuals who were selected by simple random sampling method among persons who accompanied patients referred to the vaccination and dental clinics of a public hospital (Shariati Hospital) in Tehran, Iran. The eligibility criteria consisted of being aged 30–49 years, and having no history of cardiovascular disease, diabetes, cancer, stroke, kidney diseases, and gout.

From selected individuals, 4 who had heart failure and kidney disease were not included to the study, and the participants consisted of 41 persons as the MetS group, and 60 as controls. MetS was defined according to the third report of the National Cholesterol Education Program Adult Treatment (NCEP) (Panel III) [11], i.e. having at least three of the following components: high serum triglycerides (TG) concentrations (≥150 mg/dl and/or use of lipid lowering medication); low serum HDL- cholesterol (HDL-C) concentrations (<40 mg/dl in men and <50 mg/dl in women); elevated blood pressure (BP) (≥130/85 mmHg and/or use of anti-hypertensive medication); abnormal glucose homeostasis (fasting plasma glucose(FPG) ≥ 100 mg/dl and/or use of insulin or oral hypoglycemic medication); and enlarged waist circumference (WC). The cutoffs for enlarged WC were set at > 89 cm in men and > 91 cm in women, based on guidelines for the First Nationwide Study of the Prevalence of Metabolic Syndrome in Iran [8]. The Ethics Committee of Endocrine and Metabolism Research Institute, Tehran University of Medical Sciences approved the study protocol, and informed written consent was obtained from all participants.

### Anthropometric and blood pressure measurement

Fat mass (FM, kg) and fat-free mass (FFM, kg) were estimated by means of bioelectrical impedance analysis (BIA; BC- 418MA, Tanita Company, USA) through the use of 8 polar electrodes. Body circumferences were measured at the umbilicus (waist circumference) and at the most prominent buttock level (hip circumference). Weight was measured using digital scales (Seca, Germany) while subjects were minimally clothed and without shoes, recorded to the nearest 100 g. Height was measured to the nearest 1 cm using a non-elastic tape meter while subjects were in a barefoot standing position, with their shoulders in a normal position. Body mass index (BMI) was calculated as weight in kilograms divided by the square of height in meters. The WC was taken halfway between the iliac crest and the lower rib margin, and hip circumference was measured at the maximal level over the great femoral trochanters, over light clothing, using an unstretched tape meter without any pressure to the body surface, and was recorded to the nearest 0.1 cm. BP was measured twice in the right arm of subjects who had been resting for at least 10 min in a seated position using a mercury sphygmomanometer.

### Laboratory measurements

A venous blood sample of participants was taken after a 12–14 h overnight fast, centrifuged within 2 h, and refrigerated at −10 °C. Total cholesterol (TC) and TG were determined enzymatically using Parsazmun kits (Tehran,Iran). HDL-C was measured similarly after precipitation with magnesium phosphotungstate. LDL-cholesterol was calculated using Friedewald’s formula. FPG was measured using the glucose oxidase method, and immunoreactive insulin (IRI) was measured by radioimmunoassay. Homeostasis Model Assessment (HOMA) was used for the assessment of insulin resistance. The HOMA index was calculated as FPG (mg/dl) × IRI (μU/ml)/405 [12]. Insulin was measured by radioimmunoassay. Uric acid level was determined on a standard auto analyzer with Uricase and reagent (Parsazmun Co.,Tehran,Iran).

### Statistical analysis

Results are expressed as mean ± standard deviation (SD). Kolmogorov Smirnov test was used to examine the normality of variables of interest. Continuous variables were compared by *T*-test. The relationship between uric acid level and other variables including MetS components were assessed by Pearson’s correlation coefficients. A logistic regression analysis was performed to examine the relationship between serum uric acid and the diagnosis of MetS. P-value less than 0.05 was considered significant. All statistical analyses were carried out using the statistical program SPSS (version 13, SPSS).

## Results

**Fig. 1:**
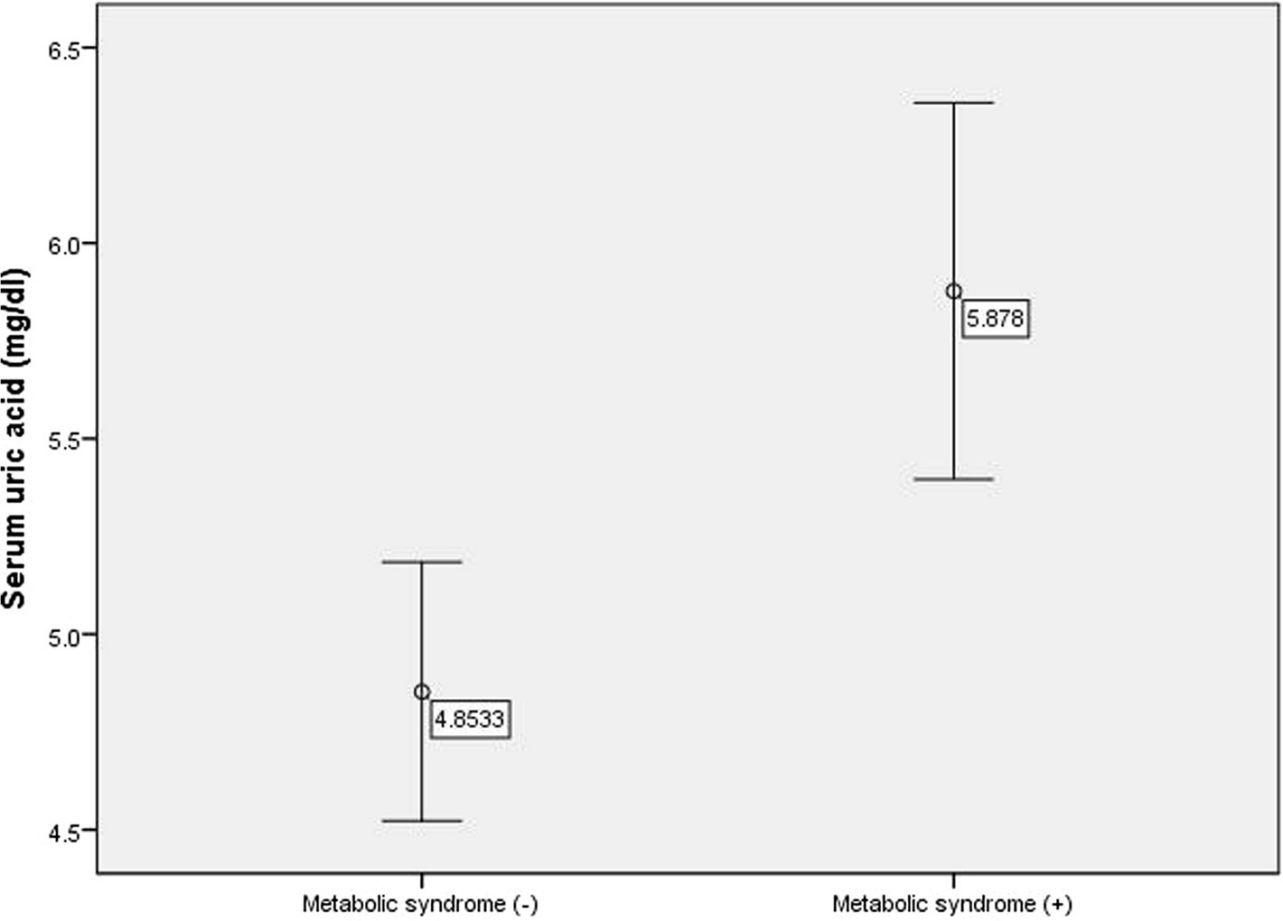
Difference in serum uric acid level in the presence of metabolic syndrome

The average age of study population was 38.5 ± 6.0 years. The clinical and metabolic characteristics of the study participants are summarized in Table 1. Subjects in the MetS group had higher BMI, WC, lean body mass (LBM), body fat mass (BFM), trunk fat mass, SBP, DBP, FPG, insulin, HOMA index, TG, TC, LDL, and lower HDL levels than the subjects in the non-MetS group. In this study, mean serum uric acid was significantly higher in MetS group than that in the non-MetS group (Fig. 1), even after adjustment for age, sex and BMI (5.70 ± 1.62 and 4.97 ± 1.30, P = 0.001 in MetS and non-MetS group, respectively).

**Table 1:** Characteristics of the participants with or without metabolic syndrome

Variables	MetS group (*n* = 41)	non-MetS group (*n* = 60)	*P*-*value*
Age, years	40.66 ± 6.01	37 ± 5.57	0.002
Body mass index, kg/m2	28.74 ± 3.73	25.79 ± 3.70	<0.001
Waist circumference, cm	95.10 ± 15.16	88.65 ± 8.58	0.011
Fat free mass, kg	55.56 ± 12.14	51.28 ± 9.28	0.048
Fat mass, kg	24.92 ± 7.6	19.26 ± 6.55	<0.001
Trunk fat mass, kg	13.37 ± 3.94	10.49 ± 3.31	<0.001
Systolic blood pressure, mmHg	122.8 ± 13.3	112.6 ± 12.5	<0.001
Diastolic blood pressure, mmHg	85.7 ± 10.3	78.3 ± 11.29	0.001
Fasting glucose, mg/dl	104.9 ± 12.1	95.8 ± 6.9	<0.001
Fasting insulin, μU/ml	12.6 ± 5.67	8.9 ± 4.36	<0.001
HOMA-IR	3.28 ± 1.49	2.12 ± 1.08	<0.001
Triglyceride, mg/dl	200.1 ± 130.3	102.4 ± 57.4	<0.001
Total cholesterol, mg/dl	200.9 ± 43.9	180.5 ± 30.6	0.007
LDL-C, mg/dl	123.8 ± 31.4	109.1 ± 24.1	0.009
HDL-C, mg/dl	42.7 ± 7.2	53 ± 11.7	<0.001
Uric Acid, mg/dl	5.87 ± 1.52	4.85 ± 1.28	<0.001

Data was expressed as mean ± SD

Table 2 shows partial correlations between serum uric acid and anthropometric and biochemical variables. Serum uric acid continued to have a significant correlation after adjusting for age and sex (model 1), except for SBP, FPG, TC and LDL-C. In this model, there were significant correlation between uric acid levels and insulin, HOMA-IR and body fat mass. After more adjustment for BMI, uric acid had significant correlation just with TGs (r = 0.322, P = 0.004) and with HDL-C (r = − 0.324, P = 0.003).

**Table 2:** Partial correlation between uric acid and other variables

Variables	Model I		Model II		Model III	
	Pearson’s coefficients	*p*-value	Pearson’s coefficients	*p-value*	Pearson’s coefficients	*p-value*
Body mass index, kg/m2	0.245	0.014	0.26	0.016	----------	---------
Waist circumference, cm	0.377	<0.001	0.24	0.027	0.078	0.492
Body fat mass, kg	0.028	0.785	0.27	0.014	0.089	0.432
Trunk fat mass, kg	0.281	0.007	0.24	0.025	0.077	0.499
Systolic blood pressure, mmHg	0.400	<0.001	0.14	0.198	0.055	0.628
Diastolic blood pressure, mmHg	0.416	<0.001	0.24	0.031	0.158	0.161
Fasting glucose, mmol/L	0.298	0.002	0.08	0.429	0.013	0.908
Insulin	0.095	0.344	0.22	0.048	0.123	0.279
HOMA	0.157	0.116	0.22	0.043	0.119	0.293
Triglyceride, (mg/dL)	0.455	<0.001	0.30	0.005	0.322	0.004
Total cholesterol, (mg/dL)	0.200	0.045	0.07	0.495	0.087	0.445
LDL-C,(mg/dL)	0.213	0.032	0.11	0.316	0.086	0.449
HDL-C, (mg/dL)	−0.454	<0.001	−0.372	0.001	−0.324	0.003

Model I: Crude model; Model II: Adjusted for age, gender; Model III: Adjusted for age, gender & BMI

Table 3 shows the association of serum uric acid level for the diagnosis of MetS in the logistic regression analysis. There were increases in ORs after adjustment for age and gender. The result of regression model showed that in model III (age, sex and BMI adjusted) for every 1 mg/dl elevation in the serum uric acid level the odds ratio for developing metabolic syndrome increased approximately 2-fold (OR:2.11, 95 %CI: 1.30-3.41).

**Table 3:** Association between uric acid level *(mg/dl)* and metabolic syndrome in logistic regression models

Serum uric acid	OR (95 % CI)	*P*
Model I	1.70(1.23-2.33)	0.001
Model II	2.38 (1.49-3.81)	<0.001
Model III	2.11 (1.30-3.41)	0.002

Model I, Crude model; Model II, adjusted for age and sex; Model III, further adjusted for BMI

**Fig. 2:**
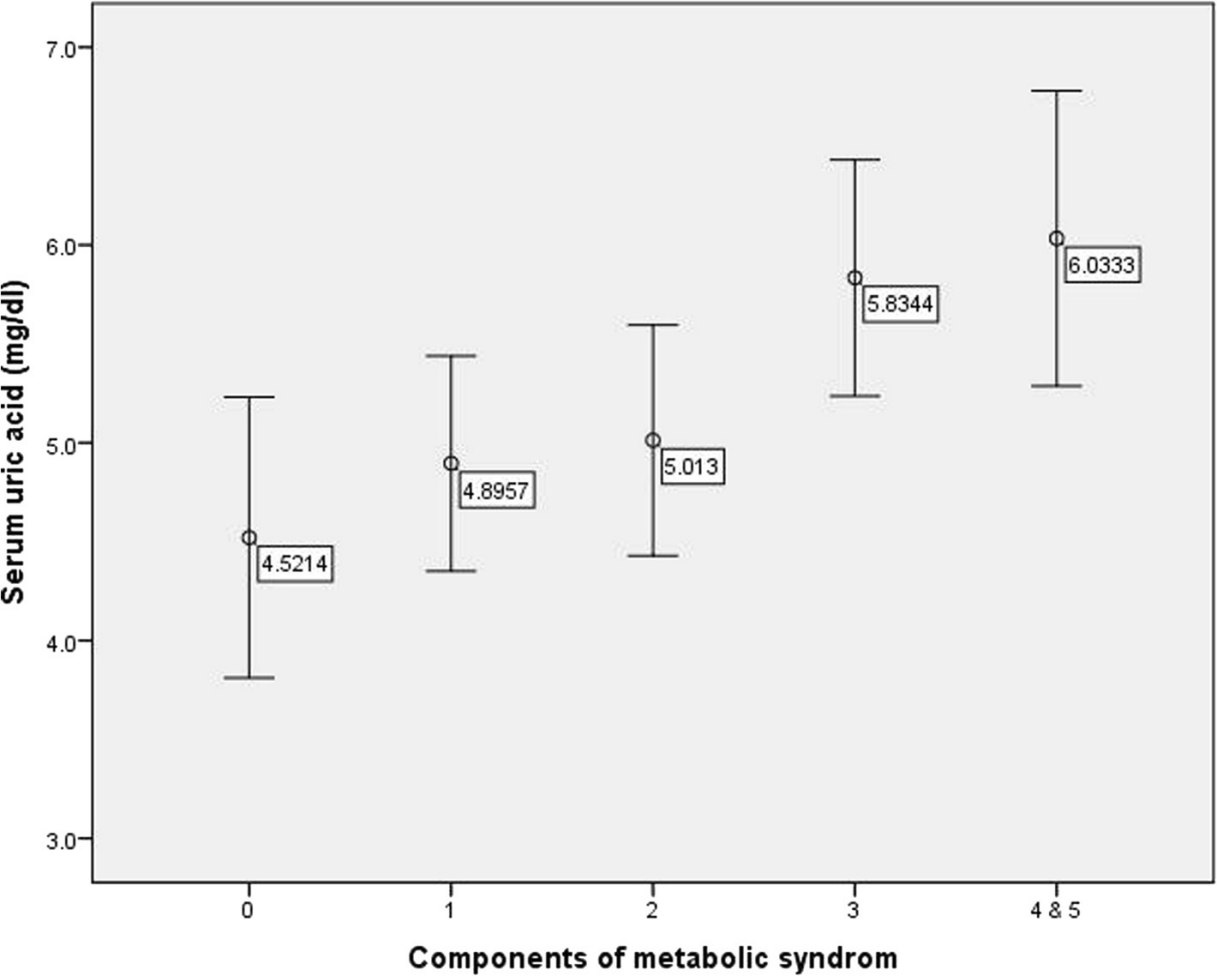
Mean and standard deviation of serum uric acid levels according to the number of metabolic syndrome components

Spearman’s correlation coefficient analysis revealed that the mean serum uric acid increased with increasing number of MetS components (r = 0.335, P =0.001) (Fig. 2). Because of few numbers of subjects with 4 and 5 MetS components and equal mean of uric acid in both groups we merged data of these participants.

## Discussion

In this study, we evaluated the associations of serum uric acid levels with MetS components in individuals with or without MetS. Serum uric acid levels were significantly higher in the MetS group compared to healthy individuals after considering covariates like gender, age and BMI. This finding is in line with some other studies [13–15]. Although hyperuricemia is well recognized as a risk factor for atherosclerotic diseases such as myocardial infarction and stroke [16, 17], its independent association with cardiometabolic risk factors remained controversial. However, Zhang et al. showed that uric acid is an independent risk factor of MetS, and its higher concentrations increased the risk of MetS [18].

We found that higher serum uric acid levels, even within the normal ranges, were associated with an increased odds ratio of MetS, and remained significant even after adjusting for confounding factors. It is speculated from this study that serum uric acid is one of the determinants of the Mets. With one unit increasing of serum uric acid, the odds of developing MetS approximately doubled. Similar findings have shown that individuals with high uric acid levels have 1.6 times higher odds of developing MetS [19, 20]. However, the precise biological mechanisms underlying the association between serum uric acid and development of MetS remain unclear, although reduction in endothelial nitric oxide bioavailability by uric acid is likely to be involved [20]. Nitric oxide seems to play an important role in the development of insulin resistance, and its deficiency is believed to reduce blood flow to insulin-sensitive tissues,i.e. skeletal muscle, liver, adipose tissue, leading to block the action of insulin [21]. Furthermore, hyperinsulinemia reduces urinary uric acid excretion by the effect of insulin on urinary tubules leading to hyperuricemia [22].

In the present study, after controlling for sex, age and BMI, serum uric acid had significant relationship with hypertriglyceridemia and low HDL-C. These findings are in line with results of some other studies [23–27]. Several past studies have shown that high level of plasma TG are related to hyperuricemia [28–30]. There are some explanations for such relation. A potential mechanism is that TG synthesis accelerates the de novo synthesis of ribose-5-phosphate to phosphoribosyl pyrophosphate (PPRP) through the common metabolic pathway of NADP-NADPH, and as a result, uric acid production increases [31]. Some previous studies reported negative correlation between HDL-C and serum uric acid [32, 33]. The mechanisms of this relation may be due to the association between decreased HDL-C levels and insulin resistance [34].

The present study showed that higher concentrations of serum uric acid were significantly associated with increased number of MetS components. This finding has been reported in some other similar studies [35–37].

It has been suggested that hyperuricemia can be an additional components of MetS. Some studies have argued that the hyperuricemia should be included as an additional MetS component [38, 39]. The results of other studies showed that serum uric acid level was associated with a higher risk of MetS across a broad age range [40, 41]. At present, hyperuricemia has not been included among NCEP criteria as a component of Mets. Our data show that serum uric acid was significantly associated with the diagnosis of MetS in the population studied, however, longitudinal studies are needed to assess whether hyperuricemia is an additional component of the MetS or not.

Our findings are, in general, concordant with results from previous reports [42–47], but can be distinguished from them in some aspects. In our participants, serum uric acid levels ranged from 2.5 to 8.9 mg/dL, whereas most previous studies have analyzed the relation between frank hyperuricemia [48–50] with MetS. Assessment of body composition and its relation with uric acid is another novel aspect of this study. Trunk fat mass had a significant correlation with serum uric acid. This significant relation was seen for both body fat mass and trunk fat mass after controlling for age and gender. These results showed that the body fat mass, especially trunk fat mass, could be related to serum uric acid. Some studies reported the relationship of body fat mass and serum uric acid. Hikita et al. reported that there were significant relations between serum uric acid and both visceral fat and total fat mass; in particular, serum uric acid was more closely related to visceral fat [37]. It is considered that insulin resistance caused by accumulation of visceral fat is the underlying mechanism.

The limitations of this study warrant consideration. Firstly, this study is limited due to its cross-sectional nature, since a causal association between uric acid and MetS could not be derived. Secondly, the sample size in this study was relatively small which could limit the generalization of our findings. It is also possible that unmeasured confounding variables may exist.

## Conclusion

Serum uric acid had independent association with MetS components, and increased the risk of MetS by near two folds. Our findings propose that uric acid can be considered as a component of MetS. Regarding high prevalence of obesity and MetS as well as the potential link between hyperuricemia and CVD, future studies should be conducted to clarify the role of uric acid in the pathogenesis of Mets and the clinical significance of the current findings.

## References

[1] Grundy SM, Brewer HB, Cleeman JI, Smith SC, Lenfant C (2004). National heart, lung, and blood institute; american heart association. Definition of metabolic syndrome: report of the national heart, lung, and blood institute/ american heart association conference on scientific issues related to definition. Circulation.

[2] Wilson PW, D'Agostino RB, Parise H, Sullivan L, Meigs JB (2005). Metabolic syndrome as a precursor of cardiovascular disease and type 2 diabetes mellitus. Circulation.

[3] Ramakrishna V, Jailkhani R (2008). Oxidative stress in noninsulin-dependent diabetes mellitus (NIDDM) patients. Acta Diabetol.

[4] Fu CC, Wu DA, Wang JH, Yang WC, Tseng CH (2009). Association of C-reactive protein and hyperuricemia with diabetic nephropathy in Chinese type 2 diabetic patients. Acta Diabetol.

[5] Guo L, Cheng Y, Wang X, Pan Q, Li H, Zhang L (2012). Association between microalbuminuria and cardiovascular disease in type 2 diabetes mellitus of the Beijing Han nationality. Acta Diabetol.

[6] Meigs JB, Mittleman MA, Nathan DM, Tofler GH, Singer DE, Murphy-Sheehy PM (2000). Hyperinsulinemia, hyperglycemia, and impaired hemostasis: The Framingham Offspring Study. JAMA.

[7] Nestel P, Lyu R, Low LP, Sheu WH, Nitiyanant W, Saito I (2007). Metabolic syndrome: recent prevalence in East and Southeast Asian populations. Asia Pac J Clin Nutr.

[8] Delavari A, Forouzanfar MH, Alikhani S, Sharifian A, Kelishadi R (2009). First nationwide study of the prevalence of the metabolic syndrome and optimal cutoff points of waist circumference in the middle east: the national survey of risk factors for non-communicable diseases of Iran. Diabetes Care.

[9] Chiou WK, Wang MH, Huang DH, Chiu HT, Lee YJ, Lin JD (2010). The relationship between serum uric acid level and metabolic syndrome: differences by sex and age in Taiwanese. J Epidemiol.

[10] Ruggiero C, Cherubini A, Ble A, Bos AJ, Maggio M, Dixit VD (2006). Uric acid and inflammatory markers. Eur Heart J.

[11] Genuth S, Alberti KG, Bennett P, Buse J, Defronzo R, Kahn R (2003). Zimmet P; expert committee on the diagnosis and classification of diabetes mellitus. Follow-up report on the diagnosis of diabetes mellitus. Diabetes Care.

[12] Matthews DR, Hosker JP, Rudenski AS, Naylor BA, Treacher DF, Turner RC (1985). Homeostasis model assessment: Insulin resistance and β-cell function from fasting plasma glucose and insulin concentrations in man. Diabetologia.

[13] Reimann M, Schutte AE, Malan L, Huisman HW, Malan NT (2008). Hyperuricaemia is an independent factor for the metabolic syndrome in a sub-Saharan African population: a factor analysis. Atherosclerosis.

[14] Sui X, Church TS, Meriwether RA, Lobelo F, Blair SN (2008). Uric acid and the development of metabolic syndrome in women and men. Metabolism.

[15] Numata T, Miyatake N, Wada J, Makino H (2008). Comparison of serum uric acid levels between Japanese with and without metabolic syndrome. Diabetes Res Clin Pract.

[16] Culleton BF, Larson MG, Kannel WB, Levy D (1999). Serum uric acid and risk for cardiovascular disease and death: the Framingham Heart Study. Ann Intern Med.

[17] Lehto S, Niskanen L, Ronnemaa T, Laakso M (1998). Serum uric acid is a strong predictor of stroke in patients with non-insulin-dependent diabetes mellitus. Stroke.

[18] Zhang Q, Zhang C, Song X, Lin H, Zhan D, Meng W (2012). A longitudinal cohort based associationstudy between uric acid level and metabolic syndrome in Chinese Hanurban male population. BMC Public Health.

[19] Chen LY, Zhu WH, Chen ZW, Dai HL, Ren JJ, Chen JH (2007). Relationship between hyperuricemia and metabolic syndrome. J Zhejiang Univ Sci B.

[20] Gersch C, Palii SP, Kim M, Angerhofer A, Johnson RJ, Henderson GN (2008). Inactivation of nitric oxide by uric acid. Nucleosides Nucleotides Nucleic Acids.

[21] Vincent MA, Barrett EJ, Lindner JR, Clark MG, Rattigan S (2003). Inhibiting NOS blocks microvascular recruitment and blunts muscle glucose uptake in response to insulin. Am J Physiol Endocrinol Metab.

[22] Heinig M, Johnson RJ (2006). Role of uric acid in hypertension, renal disease, and metabolic syndrome. Cleve Clin J Med.

[23] Nagahama K, Iseki K, Inoue T, Touma T, Ikemiya Y, Takishita S (2004). Hyperuricemia and cardiovascular risk factor clustering in a screened cohort in Okinawa, Japan. Hypertens Res.

[24] Nakanishi N, Okamoto M, Yoshida H, Matsuo Y, Suzuki K, Tatara K (2003). Serum uric acid and risk for development of hypertension and impaired fasting glucose or Type II diabetes in Japanese male office workers. Eur J Epidemio.

[25] Krishnan E, Kwoh CK, Schumacher HR, Kuller L (2007). Hyperuricemia and incidence of hypertension among men with-out metabolic syndrome. Hypertension.

[26] Coutinho Tde A, Turner ST, Peyser PA, Bielak LF, Sheedy PF, Kullo IJ (2007). Associations of serum uric Acid with markers of inflammation, metabolic syndrome, and subclinical coronary atherosclerosis. Am J Hypertens.

[27] Bonora E, Kiechl S, Willeit J, Oberhollenzer F, Egger G, Targher G (1998). Prevalence of insulin resistance in metabolic disorders: the Bruneck Study. Diabetes.

[28] Clausen JO, Borch-Johnsen K, Ibsen H, Pedersen O (1998). Analysis of the relationship between fasting serum uric acid and the insulin sensitivity index in a population-based sample of 380 young healthy Caucasians. Eur J Endocrinol.

[29] Conen D, Wietlisbach V, Bovet P, Shamlaye C, Riesen W, Paccaud F (2004). Prevalence of hyperuricemia and relation of serum uric acid with cardiovascular risk factors in a developing country. BMC Public Health.

[30] Schachter M (2005). Uric acid and hypertension. Curr Pharm Des.

[31] Matsuura F, Yamashita S, Nakamura T, Nishida M, Nozaki S, Funahashi T (1998). Effect of visceral fat accumulation on uric acid metabolism in male obese subjects: visceral fat obesity is linked more closely to overproduction of uric acid than subcutaneous fat obesity. Metabolism.

[32] Silva HA, Carraro JC, Bressan J, Hermsdorff HH. Relation between uric acid and metabolic syndrome insubjects with cardiometabolic risk. Einstein (Sao Paulo). 2015;13:202–8. 10.1590/S1679-45082015AO3194 PMC4943810 26018145

[33] Rho YH, Choi SJ, Lee YH, Ji JD, Choi KM, Baik SH (2005). The prevalence of metabolic syndrome in patients with gout: a multicenter study. J Korean Med Sci.

[34] Schmidt MI, Watson RL, Duncan BB, Metcalf P, Brancati FL, Sharrett AR (1996). Clustering of dyslipidemia, hyperuricemia, diabetes, and hypertension and its association with fasting insulin and central and overall obesity in a general population. Atherosclerosis Risk in Communities Study Investigators. Metabolism.

[35] Conen D, Wietlisbach V, Bovet P, Shamlaye C, Riesen W, Paccaud F (2004). Prevalence ofhyperuricemia and relation of serum uric acid with cardiovascular risk factors in a developing country. BMC Public Health.

[36] Desai MY, Santos RD, Dalal D, Carvalho JA, Martin DR, Flynn JA (2005). Relation of serum uric acid with metabolic risk factors in asymptomatic middle-aged Brazilian men. Am J Cardiol.

[37] Hikita M, Ohno I, Mori Y, Ichida K, Yokose T, Hosoya T (2007). Relationship between hyperuricemia and body fat distribution. Intern Med.

[38] Liou TL, Lin MW, Hsiao LC, Tsai TT, Chan WL, Ho LT (2006). Is hyperuricemia another facet of the metabolic syndrome?. J Chin Med Assoc.

[39] Sheu WH, Tseng YH (2006). Uric acid: an additional component of metabolic syndrome?. J Chin Med Assoc.

[40] Ryu S, Song J, Choi BY, Lee SJ, Kim WS, Chang Y (2007). Incidence and risk factors for metabolic syndrome in Korean male workers, ages 30 to 39. Ann Epidemiol.

[41] Sui X, Church TS, Meriwether RA, Lobelo F, Blair SN (2008). Uric acid and the development of metabolic syndrome in women and men. Metabolism.

[42] Ishizaka N, Ishizaka Y, Toda E, Nagai R, Yamakado M (2005). Association between serum uric acid, metabolic syndrome, and carotid atherosclerosis in Japanese individuals. Arterioscler Thromb Vasc Biol.

[43] Lim JH, Kim YK, Kim YS, Na SH, Rhee MY, Lee MM (2010). Relationship between serum uric acid levels, metabolic syndrome, and arterial stiffness in Korean. Korean Circ J.

[44] Lin SD, Tsai DH, Hsu SR (2006). Association between serum uric acid level and components of the metabolic syndrome. J Chin Med Assoc.

[45] Kim SK, Park HA, Nam OY, Beck SH, Whang DH, Hwang UK (2007). Risk of the metabolic syndrome according to the level of the uric acid. J Korean Acad Fam Med.

[46] Choi HK, Ford ES (2007). Prevalence of the metabolic syndrome in individuals with hyperuricemia. Am J Med.

[47] Onat A, Uyarel H, Hergenc G, Karabulut A, Albayrak S, Sari I (2006). Serum uric acid is a determinant of metabolic syndrome in a population-based study. Am J Hypertens.

[48] Feig DI, Johnson RJ (2003). Hyperuricemia in childhood primary hypertension. Hypertension.

[49] Niskanen LK, Laaksonen DE, Nyyssonen K, Alfthan G, Lakka HM, Lakka TA (2004). Uric acid level as a risk factor for cardiovascular and all-cause mortality in middle-aged men: a prospective cohort study. Arch Intern Med.

[50] Nakagawa T, Tuttle KR, Short RA, Johnson RJ (2005). Hypothesis: fructose-induced hyperuricemia as a causal mechanism for the epidemic of the metabolic syndrome. Nat Clin Pract Nephrol.

